# Supplementary material for: The genetics of extrinsic postzygotic selection in a migratory divide between subspecies of the Swainson’s thrush
Source: Nat Commun. 2025 Aug 24;16:7897. doi: 10.1038/s41467-025-63188-6 (PMC12375019; doi:10.1038/s41467-025-63188-6)
Supplement: Supplementary file 1 — Supplementary Information [file 41467_2025_63188_MOESM1_ESM.pdf]

SUPPLEMENTARY INFORMATION

The genetics of extrinsic postzygotic selection in a migratory divide between subspecies of the Swainson's thrush

Authors: Hannah C. Justen[1]*, Stephanie A. Blain[2], Kira E. Delmore[2]

[1] Neurobiology, Physiology and Behavior Department, University of California, Davis, CA, USA

[2] Ecology and Evolutionary Biology Department, Columbia University, New York City, USA

*Corresponding author, hjusten@ucdavis.edu

1. Identify sites fixed between reference genomes.

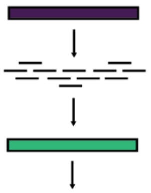

simulate reads from coastal genome

align reads to inland genome

identify sites fixed for alternate alleles

chr	pos
1	1331
1	4000
1	5467
1	9987

2. Identify sites differentiated between subspecies using reference panels.

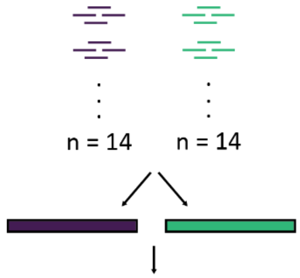

align reads from pure coastal and inland birds to both genomes

remove non-uniquely mapping reads and identify sites with allele frequency differences greater than 0.5

chr	pos	af_diff
1	1331	0.3
1	4000	0.7 *
1	5467	0.9 *
1	9987	0.4

3. Call ancestry informative sites in birds sequenced to low coverage.

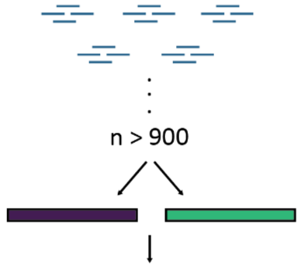

align reads from hybrid birds to both genomes

remove non-uniquely mapping reads and count reads at ancestry informative sites

chr	pos	counts coastal	counts inland
1	4000	2	1
1	5467	1	0

run HMM

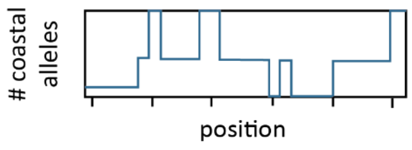

homozygous coastal

heterozygous

homozygous inland

Figure S1. Diagram to show the main steps to identify ancestry informative markers and genotype birds (i.e., assign ancestry states) at these markers.

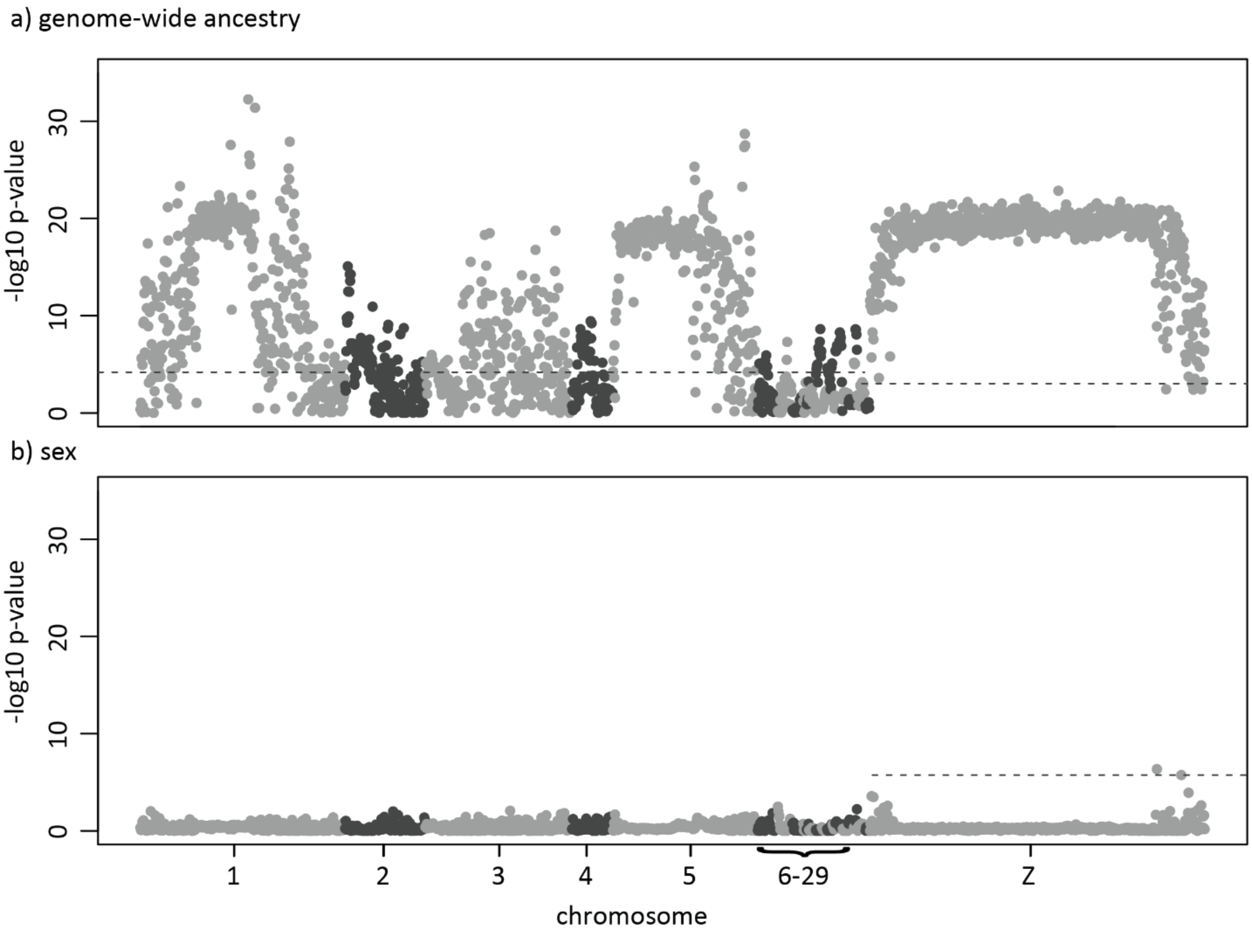

Figure S2. Viability selection and interspecific heterozygosity. Results from general linear models run for each ancestry informative marker along the genome for co-variables (a) genome-wide ancestry and (b) sex. Separate corrections for multiple testing were applied to autosomes and the Z chromosome (dotted lines indicated FDR adjusted two-sided p-values < 0.05). Shading reflects alternating chromosomes.

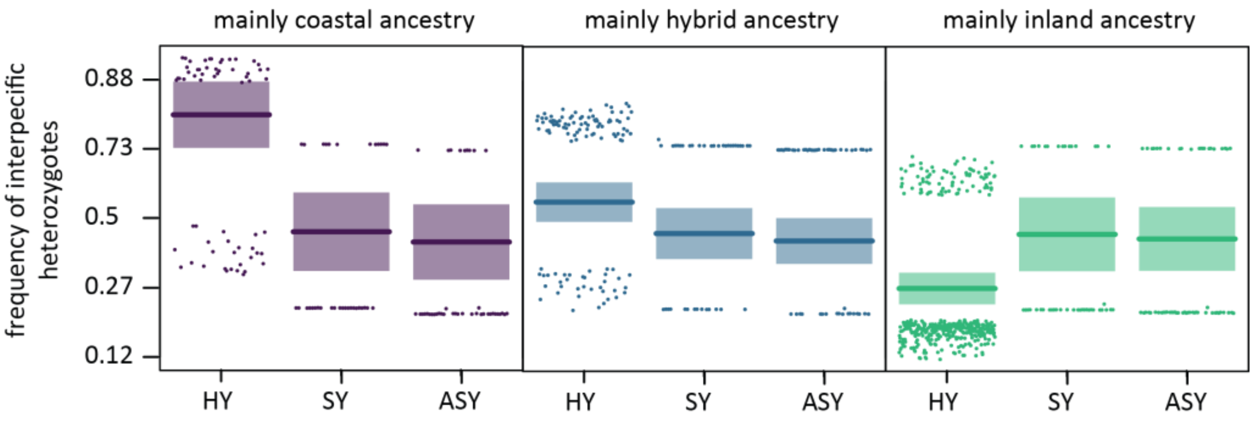

Figure S3. Viability selection and interspecific heterozygosity. Panels show change in interspecific heterozygosity across age at the locus with the strongest association on chromosome 5 separately for each ancestry group (birds with mainly coastal, hybrid and inland ancestry respectively). Predictions lines and confidence intervals are shown; colors reflect birds genotyped as mostly pure (purple and green) or hybrid (blue).

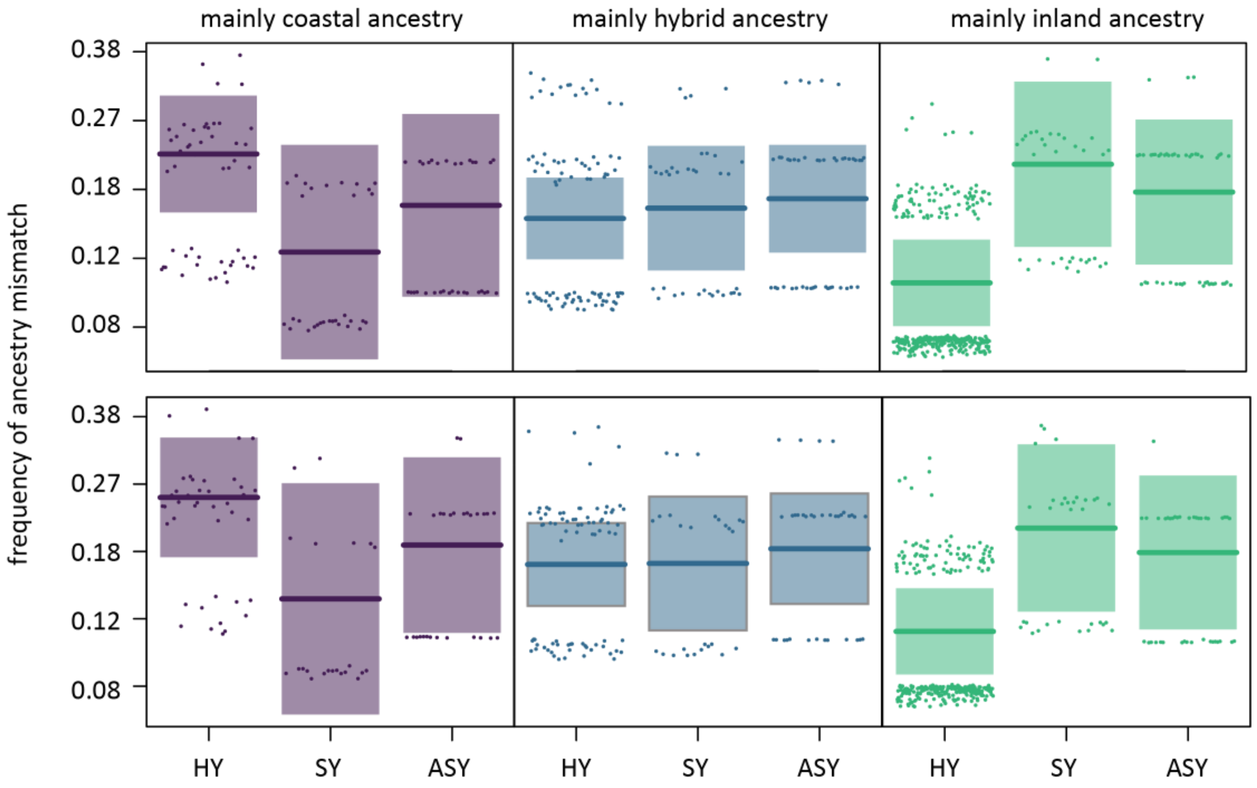

Figure S4. Viability selection and ancestry mismatch. Panels in each row show the change in ancestry mismatch across age classes separately for each ancestry group (birds with mainly coastal, hybrid and inland ancestry respectively). Loci presented showed the strongest association on chromosome 1 and the Z (top) and chromosome 5 and the Z (bottom). Predictions lines and confidence intervals are shown; colors reflect birds genotyped as mostly pure (purple and green) or hybrid (blue).

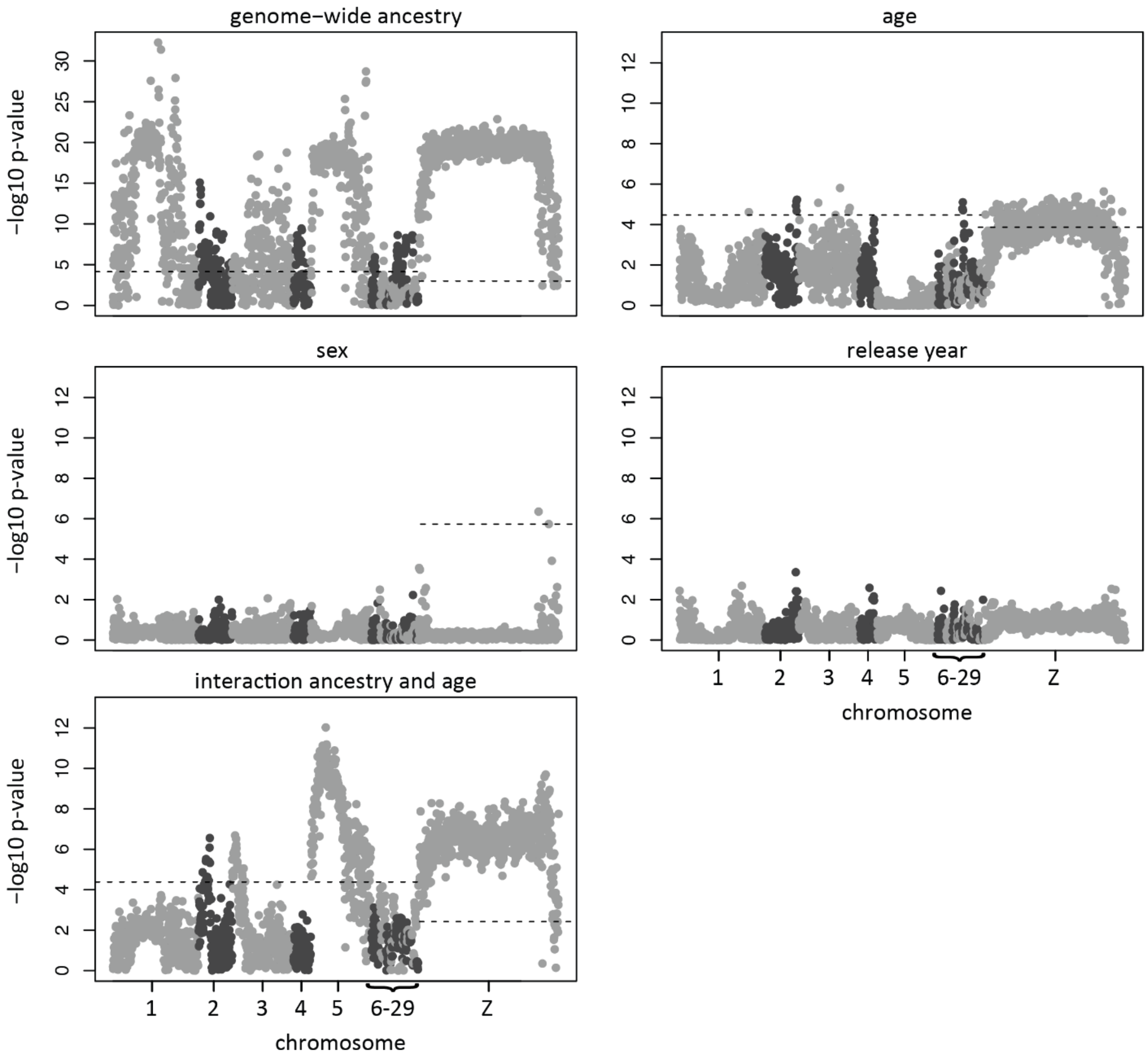

Figure S5. Alternative model - viability selection and interspecific heterozygosity. Results from general linear models run for each ancestry informative marker along the genome including release year as an additional co-variate [glm(heterozygosity at ancestry informative marker locus~ genome-wide ancestry * age + sex + release year, family=binomial(link="logit"))]. Panels show results for each variable in the model. Separate corrections for multiple testing were applied to autosomes and the Z chromosome (dotted lines indicated FDR adjusted two-sided p-values < 0.05). Shading reflects alternating chromosomes.

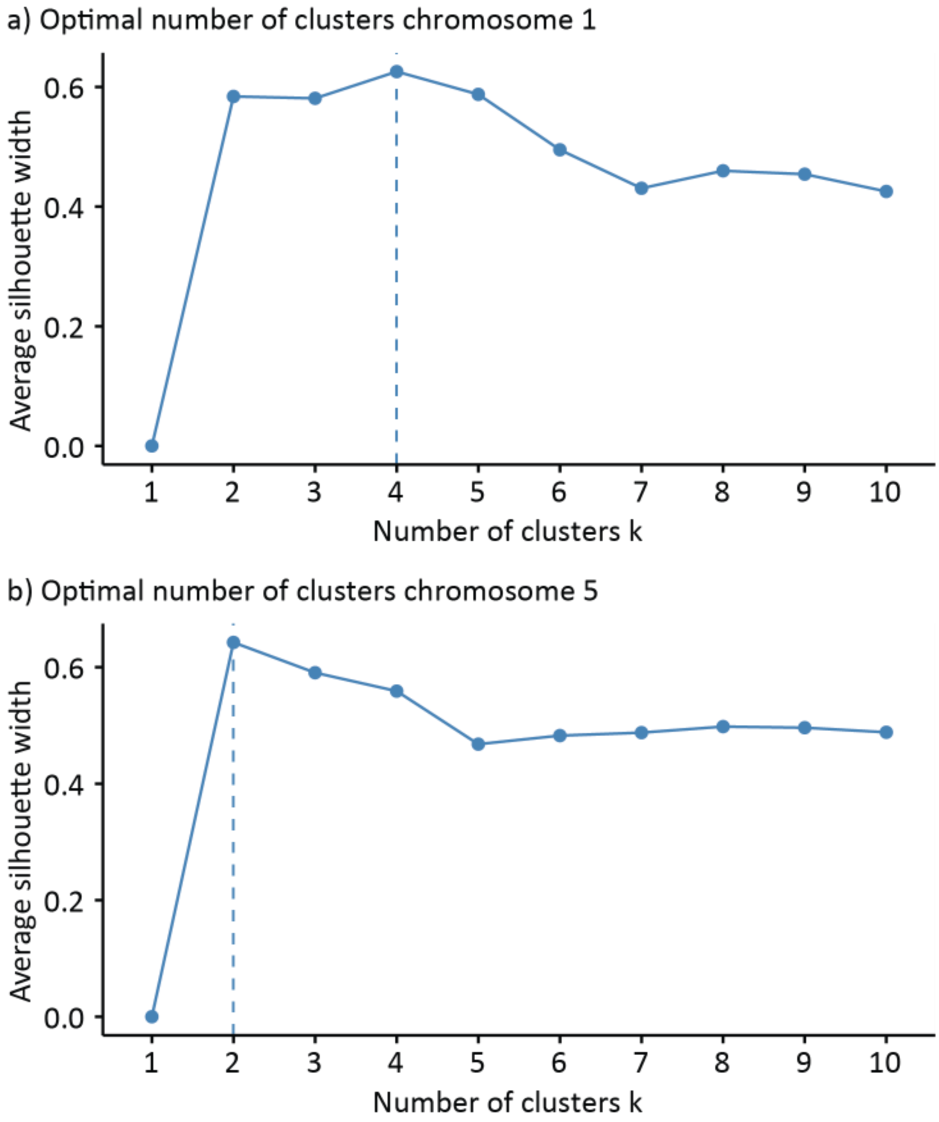

Figure S6. Silhouette analysis to identify the optimal clustering [k] for local pca analyses. (a) analysis for chromosome 1, determining 4 to be the optimal k. (b) analysis for chromosome 5, determining 2 to be the optimal k.

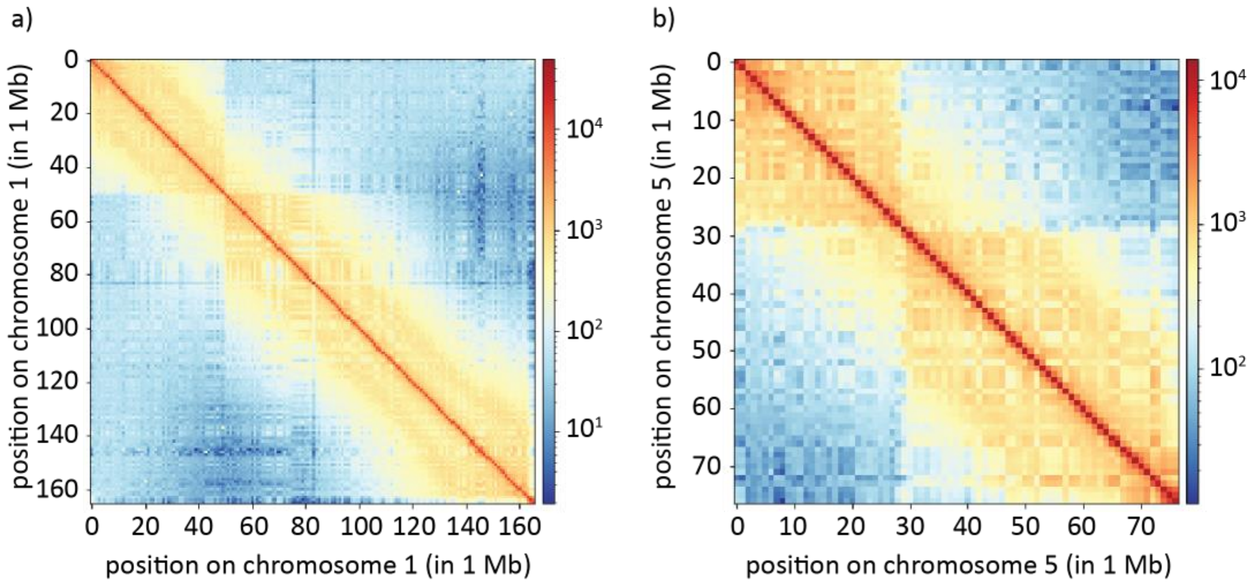

Figure S7. Hi-C data from the inland genome. (a) chromosome 1 showing centromere at 50 Mb and (b) chromosome 5 showing the centromere at 28 Mb. Red indicates elevated long-distance interactions; blue decreased long-distance interactions.
