# Supplementary material for: The genetics of extrinsic postzygotic selection in a migratory divide between subspecies of the Swainson’s thrush
Source: Nat Commun. 2025 Aug 24;16:7897. doi: 10.1038/s41467-025-63188-6 (PMC12375019; doi:10.1038/s41467-025-63188-6)
Supplement: Supplementary file 4 — Description of Additional Supplementary Files [file 41467_2025_63188_MOESM4_ESM.pdf]

Description of Additional Supplementary File

File Name: Supplementary_Data_1

Description: List of candidate genes for migratory behavior identified in previous studies (Justen et al 2024, PNAS and Louder et al 2024, Nat Commun) compared to genes significantly associated with viability selection identified in this study.
